# How energy and chemistry converge for a fossil-free future

**DOI:** 10.1016/j.isci.2025.113787

**Published:** 2025-10-17

**Authors:** Jan Mertens, Christian Breyer, Ronnie Belmans, Corinne Gendron, Patrice Geoffron, Carolyn Fischer, Elodie Du Fornel, Olivier Ledent, Richard Lester, Kimberly A. Nicholas, Laura Megrelis, Paulo Emilio Valadão de Miranda, Celine Paton, Alice Prudhomme, Peter Verwee, Olivier Sala, Michael Webber, Koenraad Debackere

**Affiliations:** 1 ENGIE Research and Innovation, 1 pl. Samuel de Champlain, 92930 Paris-la Défense, Paris, France; 2 Department of Electromechanical, System and Metal Engineering, Ghent University, Technologiepark Zwijnaarde 131, Zwijnaarde, Belgium; 3 LUT University, Yliopistonkatu 34, 53850 Lappeenranta, Finland; 4 Electrical Energy and Computer Architectures, K.U.Leuven, Kasteelpark Arenberg, 3001 Leuven, Belgium; 5 EnergyVille, Thor Park 8310, 3600 Genk, Belgium; 6 Université du Québec à Montréal (UQAM), département de Stratégie, Responsabilité sociale et environnementale, École des sciences de la gestion (ESG), Montréal, QC, Canada; 7 Dauphine Economics Laboratory, Université Paris Dauphine-PSL, Place du Maréchal de Lattre de Tassigny, 75016 Paris, France; 8 Development Research Group, World Bank Group, 1818 H Street, NW, Washington, DC 20433, USA; 9 School of Business and Economics, VU-Amsterdam, De Boelelaan 1105, 1081 HV Amsterdam, the Netherlands; 10 ENGIE S&EM Market Analysis, Simon Bolivarlaan 34, 1000 Brussel, Belgium; 11 Department of Nuclear Science and Engineering, Massachusetts Institute of Technology (MIT), Cambridge, MA, USA; 12 Lund University Centre for Sustainability Studies (LUCSUS), Lund University, 221 00 Lund, Sweden; 13 Hydrogen Laboratory at Coppe-Federal University of Rio de Janeiro, Av. Moniz Aragão, 207, Rio de Janeiro 21941-594, Brazil; 14 Department of Mechanical Engineering, The University of Texas at Austin, 204 E. Dean Keeton St., Stop C2200, Austin, TX 78712-1591, USA; 15 KU Leuven, ECOOM, Department of Managerial Economics, Strategy and Innovation, Faculty of Economics and Business, Naamsestraat 69, 3000 Leuven, Belgium

**Keywords:** earth sciences, energy resources, energy policy, energy sustainability, energy systems

## Abstract

The chemical industry must undergo a dual transformation: electrifying energy use and defossilizing carbon feedstocks. This paper, developed by ENGIEs Scientific Council, examines how energy and chemistry can converge to enable this shift. We assess the roles of biomass, recycled plastics, and CO_2_ as sustainable carbon sources and explore the enabling potential of electrification, low-carbon hydrogen, and direct air capture. Novel process pathways and infrastructure scenarios are analyzed to highlight strategic opportunities for cross-sectoral collaboration. Our findings underscore the need for coordinated investment, policy support, and alignment with renewable energy geography to achieve a resilient, fossil-free future.

## Graphical abstract

**Figure:**
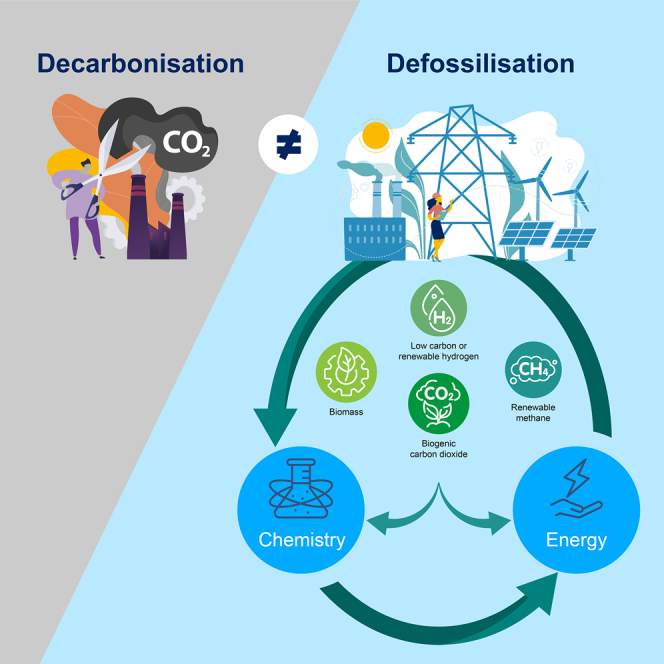

## Introduction: Defossilization rather than decarbonization

The chemical industry is foundational to modern economies and societies, underpinning broad economic sectors ranging from agriculture and construction to healthcare and consumer goods. However, the industry is also among the most carbon-intensive human activities, contributing to approximately 4% of the global CO_2_ emissions.^1^ As many chemicals are derived from carbon-based feedstocks, the challenge for the chemical industry is not to decarbonize but to “defossilize,” using both renewable energy and sustainable carbon sources as a feedstock.

The urgency to defossilize is growing, driven by climate commitments and increasing societal pressure for sustainable production models. The pressure for the chemical industry, in general, is to reach carbon neutrality for which defossilization is one of the avenues. One may question whether the option of continuing the use of fossil feedstock and compensating by negative emissions (carbon dioxide removal [CDR]) elsewhere is more cost-effective than biobased molecules or carbon capture and utilization, which require the use of non-fossil hydrogen combined with a sustainable carbon source. Bioenergy with carbon capture and storage (BECCS) faces sustainability challenges if deployed at the volumes that will be required, and although no CDR technology is without challenges and drawbacks, direct air carbon capture and storage (DACCS) is promising.^2^ However, at present, both from a technical and an economic perspective, compensation with BECCS and DACCS is not a viable alternative and may not be so in the foreseeable future. Moreover, the Intergovernmental Panel on Climate Change (IPCC)^3^ emphasizes that deep, rapid, and sustained emission reductions are the priority and that CDR should be deployed primarily to counterbalance hard-to-abate residual emissions or to address overshoot, rather than as a substitute for mitigation. In line with this, the European Commission has proposed a 2040 target of a 90% reduction in net greenhouse gas emissions relative to 1990 levels as an interim step toward climate neutrality, with removals explicitly intended only to balance remaining unavoidable emissions.^4^ Both IPCC assessments and European Union (EU) policy frameworks, therefore, treat removals as complementary to, rather than as a replacement for, emission reductions and explicitly caution against their use as an excuse to prolong fossil fuel use. Ultimately, the combination of technology readiness and economic viability will be the judge of the dominant design that will emerge, while the aim of our study is to study dominant designs that avoid the persistent use of fossil fuels, even when compensated for.

At the same time, the global energy system is undergoing a profound transition. The shift toward renewable electricity, green molecules, and circular resource flows is reshaping value chains in both the chemical and energy sectors.^5^^,^^6^ For the chemical sector, this dual transition—of both energy and carbon feedstocks—presents a complex challenge: how to maintain competitiveness and product performance, while phasing out fossil-based inputs, both at energy and feedstock levels. The context of the chemical sector in Europe is particularly challenging because of high energy prices, tightening environmental regulations, and global overcapacity inducing fierce competition. At the same time, the urgency to act mounts. Climate targets, such as the EU’s Fit for 55 and the global push for net-zero emissions by mid-century^3^ are placing transformational pressures on the chemical industry. Despite its critical role, the sector has received comparatively limited policy support. This asymmetry risks delaying innovation and investment in sustainable chemical pathways.

**Figure 1:**
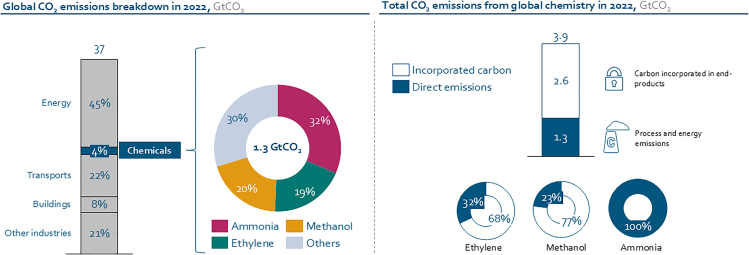
The chemical sector’s direct emissions account for around 4% of global CO_2_ emissions or 1.3 GtCO_2_ (IEA, 2023), of which ammonia, methanol, and ethylene production take a 70% share^12^ These direct emissions are only one-third of the total carbon footprint of the sector, with two-thirds incorporated in the end products referred to as downstream scope 3 emissions.^7^

While energy-intensive sectors such as power generation and transport have begun to “decarbonize’’ through electrification and renewable electricity integration via grid adjustments and storage expansion, the chemical industry faces a more complex challenge: its carbon footprint is not only energy related but also embedded in the molecular structure of its products. Based on its energy and process purposes, the sector emits an equivalent of 1.3 Gt of CO_2_.^7^ This is only one-third of its CO_2_ footprint, though. The sector uses fossil hydrocarbons, i.e., oil, gas, and coal, as feedstock source of carbon and hydrogen to produce chemicals and plastics. As depicted in Figure 1, the carbon incorporated in the sector’s end products is double its direct emissions, leading to an additional amount of carbon equivalent to 2.6 Gt of CO_2_ annually.^7^ This implies that “decarbonization” is not possible since carbon is an essential feedstock of chemicals, plastics, and materials. Defossilization of the chemical industry is not only a more appropriate term for the chemical sector^8^ but also particularly challenging, as it requires not only cleaner energy inputs but also a fundamental shift in feedstock sourcing and process design. Figure 1 also shows that 70% of the overall chemical sector emissions are associated with the production of only three chemicals: methanol (a building block for chemicals), ammonia (primarily used in fertilizers), and ethylene (primarily for plastic production). Encouragingly, these three chemicals can be entirely defossilized, as shown for e-ammonia,^9^ e-methanol,^10^ and e-ethylene.^11^

Greenhouse gas emissions can be categorized as scope 1 (direct emissions from owned or controlled sources), scope 2 (indirect emissions from purchased energy), and scope 3 (all other indirect emissions across the value chain). Companies are forced to rethink their strategies, not only to reduce scope 1 and 2 emissions through electrification and process innovation but also to address scope 3 emissions embedded in their products. In the case of chemicals, two-thirds of their emissions occur downstream. Reducing total emissions requires a systemic reconfiguration of feedstock origination and sourcing, process technologies, and supply chain logistics.

In this study, we explore how sustainable carbon feedstocks, i.e., sustainable biomass, recycled plastics, and CO_2_, can be integrated into the chemical industry to replace oil and gas and how the energy industry can play a pivotal role in enabling this transformation. The work aims to answer the following research questions:

(1) To what extent can electrification be a key lever to defossilize the chemical sector?
(2) What could be the role of hydrogen in the defossilization of the chemical industry, given its current technological and economic challenges?
(3) What are the impacts of cross-sectoral competition and the opportunities for collaboration on sustainable carbon?

To address these questions, an initial 2-day face-to-face meeting was convened in May 2024, bringing together startups, academics, chemical companies, and representatives of relevant research ecosystems for presentations, analyses, and data-driven insights, followed by in-depth question and answer sessions. Detailed analyses of the extant scientific literature on various solution avenues as highlighted above were conducted, considering both technological readiness levels of the technologies under consideration and the prevailing economic boundary conditions when analyzing forward-looking perspectives incorporating the current, varied uncertainties experienced with the various pathways. The authors are members of ENGIE’s Scientific Council, who combine academic and industrial expertise in research and development (R&D) management, energy, chemistry, environmental economics and policy, sociology, sustainability, and climate change. This implies that this work represents an expert appraisal embedded in diverse scientific backgrounds ensuring an interdisciplinary approach. Between May 2024 and January 2025, several online discussions were held with council members and ENGIE experts, both in group settings and one-on-one, to further develop and refine the inquiries. A final 2-day face-to-face meeting was held in January 2025, where the study’s outcomes were discussed, configured, and ultimately approved and supported by all the council members based on the most recent insights available from their own research and the multidisciplinary analyses of relevant scientific studies. The research design thus followed a Delphi-panel logic.

## Sustainable carbon feedstocks: availability, competition, and prioritization

Each sustainable carbon feedstock (biomass, recycled plastics, or CO_2_) presents distinct technological and economic advantages and limitations, and their relative deployment will significantly influence the feasibility, cost, and pace of defossilization. Growing demand from diverse sectors for each potential carbon feedstock source will put the chemical industry in competition with multiple other sectors that will vie for them. Estimates of their future contribution to the chemical sector vary across studies. For instance, Nova Institute (2024) projects a distribution of 25% CO_2_-based, 20% biomass-based, and 55% recycling-based chemicals and plastics. In contrast, Vogt and Weckhuysen^13^ anticipate a higher reliance on CO_2_, estimating a split of 50% CO_2_-based, 25% biomass-based, and 25% recycling-based inputs. These differences underscore the uncertainty and evolving nature of the composition of the sustainable carbon landscape, as well as the need for flexible, regionally adapted strategies.

### Biomass

Biomass is currently the best-established sustainable carbon feedstock, already integrated into various bio-based chemical processes. However, its future role in defossilizing the chemical sector is constrained by several factors: land use competition, including agriculture, sustainability criteria, and growing demand from other sectors such as aviation, maritime, power generation, heat supply, and construction. This competition puts pressure on the finite supply of sustainably sourced biomass.

Estimates of global sustainable biomass availability vary significantly depending on the geographic context and the stringency of sustainability criteria applied. Given conservative assumptions that prioritize biodiversity, food security, land-use integrity, and global availability, worldwide biomass availability is estimated to range between 50 and 100 EJ/y.^14^^,^^15^ Latest insights^16^ indicate that the upper limit of 100 EJ/y may be available from only using residues, by-products, and wastes, i.e., fully avoiding land-use conflicts of energy crops. Geographic disparities also complicate the picture. Countries like Brazil benefit from abundant biomass resources due to favorable climate and land availability conditions, whereas others, such as Belgium and the Netherlands, Singapore, or Qatar, face more constraints. These regional imbalances necessitate a differentiated approach to biomass deployment. However, those residues may also become valuable in other sectors, e.g., forestry, bringing new challenges of both the energy and chemical sectors to the forefront.

Given this limited and uneven availability, biomass should be strategically prioritized for applications where alternative decarbonization pathways are either technologically immature or economically unviable. These include sectors such as pulp and paper, chemicals and plastics, woody construction materials, and sustainable aviation fuels. In regions with more abundant biomass, additional applications may be viable, including district heating, high-temperature industrial heat, seasonal power balancing, steel production, and maritime transport. This prioritization is essential to ensure that biomass contributes effectively to climate goals without exacerbating land-use conflicts or undermining ecological integrity and biodiversity priorities.^17^

### Recycled plastics

Recycled plastics are expected to become a major pillar of sustainable carbon sourcing in the chemical and polymer industries.^18^ However, today only about 10% of plastics are recycled globally, reflecting limitations in collection systems, material sorting, and end-market demand. However, projections indicate that this share could increase to approximately 45% by 2050, provided that enabling conditions, i.e., technological, regulatory, and infrastructural, are met.^19^

Mechanical recycling is already established for certain polymers such as polyethylene terephthalate (PET) and high-density polyethylene, but its applicability is constrained by contamination, polymer degradation, and limited compatibility with mixed waste streams. Chemical recycling is still in the early stages of industrial deployment, and while it is much more flexible in terms of feedstock, it does remain energy intensive. Both approaches will need to scale significantly to meet future demand, technologically and organizationally. This growth will require coordinated action across the value chain. This includes investments in advanced sorting technologies, the expansion of chemical recycling infrastructure, and the implementation of policy instruments such as recycled content mandates. Chemical recycling processes are energy intensive, creating opportunities for energy providers to supply low-carbon power to the recycling industry. Energy providers can also explore synergies with heat integration and emerging feedstock and renewable energy-related chemical processes. Therefore, those opportunities may also present challenges and possible drawbacks since this energy intensity will also emphasize the need to prioritize energy use.

Finally, it is often assumed that all biogenic or second-life carbon is inherently “good,” yet recent analyses challenge this view. Liang et al.^20^ showed that while many biobased chemicals outperform fossil analogs, processes requiring extensive deoxygenation can exhibit higher life cycle burdens. Similarly, Uekert et al.^21^ demonstrated that not all plastic recycling routes surpass virgin petrochemical production; mechanical recycling and PET glycolysis perform best, whereas other methods may increase impacts. Together, these findings highlight that the sustainability of biogenic or recycled carbon also depends strongly on process choice rather than feedstock origin alone.

### Carbon dioxide

Carbon dioxide (CO_2_) is emerging as an important feedstock in the defossilization of the chemical industry, particularly in the context of long-term net-zero strategies. Unlike biomass or recycled plastics, CO_2_ offers the potential for near-unlimited availability, especially when captured directly from the atmosphere. However, its use as a feedstock is still in its infancy and its scaling presents both technical and even more so economic challenges.^8^ CO_2_ will be essential to close the carbon supply gap in a fully defossilized chemical sector. While biogenic CO_2_ is limited in volume, direct air capture (DAC) is expected to play a pivotal role in providing scalable, location-independent carbon inputs, underscoring the need for early investment in DAC research and technology development and elevating technology readiness levels (TRL) and economics and CO_2_ logistics infrastructure. For the foreseeable future (10–15 years) DAC will be limited in volume. Since it depends on clean energy supply, ideally available as a constant supply to keep down costs given the high CapEx of DAC, there is for now an economic and feasibility limitation to DAC, besides the scaling of DAC itself, that will have to be overcome by continuous R&D efforts. Such efforts are on their way, as our extant review of the literature shows.

The DAC cost trajectories estimated by different studies^22^^,^^23^^,^^24^^,^^25^ differ significantly, highlighting the high uncertainty (and hence still low TRL level) of DAC technology at present. However, they all show a steep decline from approximately US$400–800 per ton of CO_2_ today to projected values as low as $200 per ton by 2030 and down to US$100 per ton (or even less) by 2050. All technologies in an early industrial phase imply large uncertainties in the prediction of their future cost. Recent estimates report even wider ranges for future costs.^26^^,^^27^ It is, therefore, important that this uncertainty is integrated in the planning processes of the chemical industry. While strong growth is projected in DAC capacity, rising from less than 0.2 MtCO_2_/y in 2022 to over 1 GtCO_2_/y in 2050,^25^ actual growth is slower than projected, with current (mid-2025) installed capacity of around 0.5 Mt CO_2_, far below the projection of 6.8 MtCO_2_/y by the end of 2025.^25^ In the International Energy Agency (IEA) Net Zero Emissions by 2050 Scenario,^22^ DAC technologies capture more than 85 Mt of CO_2_ in 2030 and around 1 GtCO_2_/y in 2050. The Global CCS Institute (2024) projects 7 GtCO_2_/y of capacity at DAC costs of US$137 per ton and 2 GtCO_2_/y of capacity at DAC costs of US$237 per ton. If achieved in real-world conditions, declining costs and increasing capacity could make DAC a viable and scalable source of sustainable carbon, particularly in regions with limited access to biogenic CO_2_ or biomass. At the same time, and characteristic of the uncertainties the sector faces, Millinger et al.^28^ made the remark that when scaling DAC, the biomass-based carbon capture might be able to retain cost-competitiveness compared to DAC across a large range of uncertainties and thereby hamper the deployment of DAC. This consideration raises the concern to what extent the industry should rely on DAC to provide carbon. However, given the significant amount of research efforts pouring into DAC and the technological progress foreseen in the next decade coupled to the need for a sustainable carbon feedstock, sufficiently large-scale DAC must be considered as a viable alternative alongside sustainable biomass that will face competition for other uses as well. Hence, a co-existence seems a valuable way forward.

The production of chemicals and plastics often requires coupling CO_2_ with low-carbon hydrogen making them, e.g., in the case of green hydrogen, highly dependent on the availability of renewable electricity, hydrogen infrastructure, and robust carbon transport networks. Currently, the European Clean Industrial Deal promotes low-carbon hydrogen and not just green hydrogen, which may push this coupling.

Low-carbon hydrogen is hydrogen produced through pathways that substantially reduce life cycle greenhouse gas emissions compared to conventional fossil-based hydrogen, including renewable electrolysis (green hydrogen), fossil routes with carbon capture (blue hydrogen), and other emerging low-emission processes.

According to the IEA,^29^ the chemical sector (predominantly for ammonia) seems to be the most promising off taker for low-carbon hydrogen given the number of firm agreements already in place and the corresponding, committed large volumes. The energy intensity of these pathways underscores the importance of integrated planning across the energy and chemical sectors. Vogt and Weckhuysen^13^ estimate that a future fossil-free refinery would operate at a capacity of approximately 81,000 barrels per day (bpd), just over half the size of today’s average refinery of 150,000 bpd. This downsizing is primarily attributed to the anticipated phase-out of gasoline, assuming full electrification of road transport by 2050. Of the remaining output, around 40,500 bpd or around half would consist of kerosene jet fuel and diesel, which are still projected to be used for aviation and heavy-duty transport. Assuming these fuels are produced via Fischer-Tropsch synthesis^30^ using CO derived from DAC and green hydrogen, the process would demand a total installed renewable electricity capacity of approximately 7.3 GW.^13^ This includes 1.5 GW for DAC, 3.7 GW for hydrogen production, and 2.1 GW for CO_2_-to-CO conversion. To put this in perspective, this represents roughly 25% of Belgium’s current installed electricity generation capacity. Such a transition would not only require substantial investments in solar photovoltaics and wind power infrastructure but also significant volumes of raw materials, some of which are classified as critical by the European Union.^31^ Ensuring sustainable access to these materials is, therefore, essential. Previous work by ENGIE’s Scientific Council^2^ has outlined mitigation strategies to address potential supply chain vulnerabilities, including material efficiency, material or technology substitution, recycling, and circular design principles.

**Figure 2:**
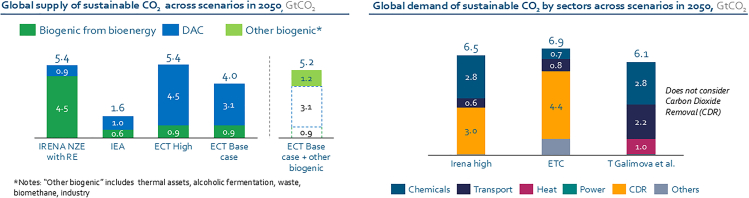
Estimated global supply and demand of sustainable CO_2_ in 2050 across multiple scenarios Supply estimates exceed predicted demand; the mismatch highlights the need for DAC to supplement biogenic sources.

As illustrated in Figure 2, in a net-zero scenario, the demand for sustainable CO_2_ is projected to significantly exceed supply, creating a gap that must be addressed to enable full defossilization. Global demand could reach up to 6.9 GtCO_2_ per year, primarily driven by the needs of carbon dioxide removal (CDR), the needs of the chemical sector, and transport applications.^32^ Notably, Galimova et al.^33^ estimate a demand exceeding 6 GtCO_2_ by 2050, even without accounting for CDR, underscoring the scale of the challenge. In contrast, the estimated supply of sustainable CO_2_ from biogenic sources ranges from as low as 0.6 GtCO_2_^22^ to a maximum of 4.5 GtCO_2_^34^ by 2050, depending on the scenario assumptions held. This results in a projected gap of several Gt, particularly in high-ambition pathways. DAC, therefore, emerges as a critical technology to close this gap, especially in sectors with a higher willingness to pay for CO_2_, such as CDR, polymer production, and construction materials. The strategic relevance of DAC is further supported by Mertens et al.,^35^ who propose a quality-based evaluation of CDR technologies, highlighting the need for DAC-CCS to evolve into a high-performing option in terms of environmental, social, and governance criteria and sequestration permanence. This strategic relevance positions CO_2_ not only as a necessary component of the sustainable carbon mix but also as a strategic lever for deep decarbonization. The successful deployment of DAC, and potentially direct ocean capture, will depend on further R&D evolutions and accelerated technological development, access to sufficient volumes of renewable electricity, low-carbon hydrogen, and the establishment of robust and economical infrastructures for CO_2_ capture, purification, and distribution. For energy providers, this represents a pivotal opportunity to support the emergence of a circular carbon economy centered on CO_2_ valorization.

## Novel processes and pathways to produce sustainable chemicals

In this section, we highlight some novel processes that are under development to produce sustainable chemicals. Perhaps the most prominent and technologically mature pathway for producing e-chemicals from renewable sources involves the synthesis of platform molecules using green hydrogen (produced with renewable energy) and carbon dioxide captured directly from the atmosphere.^5^^,^^36^ This class of technologies, which couples water electrolysis with DAC and subsequent catalytic conversion, has already been addressed in detail in earlier work, where its techno-economic performance, integration challenges, and strategic relevance were thoroughly assessed.^8^^,^^37^ The objective of this study is not to be exhaustive but to highlight pathways with an immediate link between the energy and the chemical industry.

### High-temperature heat through electrification

The decarbonization of high-temperature industrial processes is increasingly supported by the emergence of electric heating technologies, which start offering a viable alternative to fossil fuel-based thermal energy when renewable or low-carbon electricity is used. High-temperature heat pumps are under development, but their temperature level is not likely to exceed 180°C–200°C, which is insufficient for many processes in the chemical industry. However, their deployment in other industries requiring lower heat levels such as the food, textile, and tobacco industries is kicking off today. For higher-temperature heat (up to 1,000°C–2,000°C or more) technologies such as electric boilers, resistance and induction heating, plasma torches, and electric arc furnaces are reaching high(er) TRLs (TRL 7–9), with some already deployed at commercial scale.^38^ While resistance and induction heating offer high thermal efficiencies (up to 98%), emerging technologies, such as shockwave heating, promise further gain in product yield and selectivity. The challenge for the electrification of the chemical industry is, therefore, no longer related to a lack of high-temperature heat technologies. However, their large-scale deployment will depend on the availability of affordable, low-carbon electricity and the expansion of grid infrastructure to accommodate gigawatt-scale industrial loads, including economically viable OpEx and CapEx boundary conditions. For instance, a single electric steam cracker requires approximately 0.8 GW of power (size of a standard nuclear reactor), and with around 40 such units in the EU, the cumulative impact on the electricity grid could be substantial. Again, these developments underscore the need for coordinated investment in both technology and energy systems to enable the electrification of heat-intensive chemical processes.^39^^,^^40^

### Low-carbon hydrogen integration into biomass to chemical pathways

The integration of hydrogen into biomass conversion processes^41^ represents a promising strategy to enhance carbon efficiency in the production of high-value chemicals. Biomass is inherently hydrogen lean and oxygen rich, whereas most chemical products are hydrogen rich. This elemental mismatch leads to significant carbon losses during conventional thermochemical conversion. By introducing hydrogen from low-carbon sources into the conversion pathway, the carbon yield of biomass-to-chemical processes can be substantially increased. This approach not only improves the overall atom economy but also reduces CO_2_ emissions by minimizing the formation of CO_2_ as a by-product. As illustrated in recent process simulations,^41^ hydrogen-assisted biomass valorization can significantly shift the carbon balance toward target molecules, thereby supporting the dual objectives of defossilization and resource efficiency. This strategy highlights the synergistic role of low-carbon hydrogen in enabling circular carbon flows within the chemical industry.

### Novel pathways to convert syngas/biogas/biomethane to chemicals and plastics

Biogas and biomethane represent promising renewable feedstocks for the production of chemicals and plastics, offering a viable pathway to defossilize the chemical sector. These molecules can be converted into syngas (CO + H_2_), a versatile intermediate used in the synthesis of fuels, methanol, ammonia, and high-value chemicals. Steam methane reforming, the most mature technology (TRL 9), is widely used today but emits significant CO_2_ and requires water. Emerging alternatives such as dry methane reforming and super dry methane reforming use CO_2_ instead of H_2_O, improving carbon efficiency and eliminating the need for water input. For example, super dry methane reforming follows the reaction CH_4_ + 3CO_2_ → 4CO + 2H_2_O, producing a CO-rich syngas ideal for Fischer-Tropsch synthesis. These routes are particularly attractive when using biogenic CO_2_ from anaerobic digestion, enabling a more circular carbon economy and reducing net emissions.^42^

Apart from going via the syngas pathway, direct conversion technologies are being developed to transform biomethane into olefins, the key precursors for plastics. Oxidative coupling of methane (OCM) and non-oxidative coupling of methane (NOCM) are two such pathways. As indicated in Table 1, OCM, operating at 700°C–900°C with oxide catalysts (e.g., La_2_O_3_) enables the partial oxidation of methane to ethylene and ethane, with higher selectivity due to controlled oxidation. NOCM, in contrast, operates at >1,000°C without an oxidant and produces solid carbon as a by-product, avoiding CO_2_ emissions but facing challenges such as coking and catalyst deactivation. These technologies are currently at TRL 4–7 levels and require further R&D to improve selectivity and process stability.^43^^,^^44^^,^^45^

**Table 1:** Comparative analysis of NOCM and OCM as emerging pathways for direct methane-to-olefin conversion

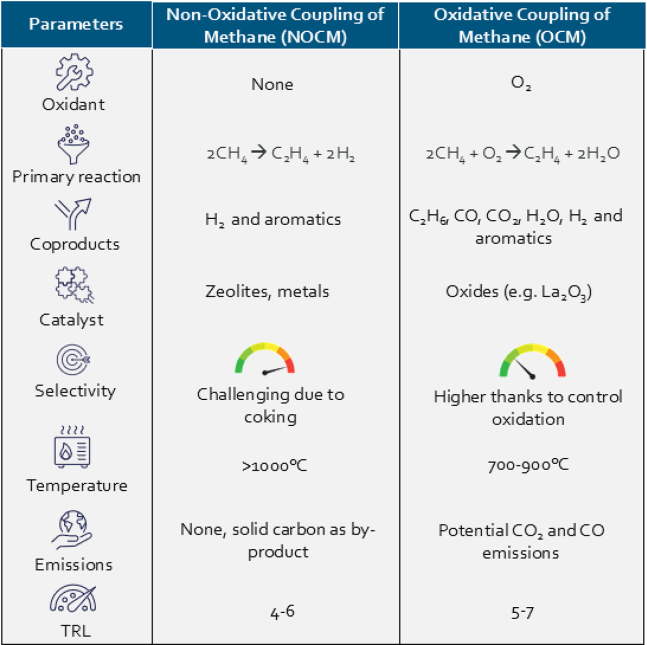

The strategic value of biomethane lies not only in its renewable origin but also in its compatibility with existing infrastructures. It can be injected into the gas grid and used as a drop-in replacement for fossil methane in existing methanol and ammonia plants, reducing the need for new capital investments. Given that methanol and ammonia together account for over 300 Mt of annual production globally, even partial substitution with biomethane could yield significant emission reductions. Moreover, the use of biomethane-derived syngas in Fischer-Tropsch synthesis or methanol-to-olefins processes could enable the production of sustainable plastics and fuels at scale. However, economic viability will depend on biomethane availability, cost competitiveness, and the development of robust CO_2_ capture and hydrogen supply chains to support these conversion routes.

## Sustainable carbon and hydrogen logistics: Where to do what?

**Figure 3:**
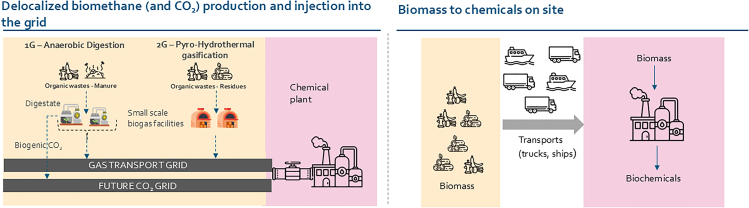
Comparison of two pathways for integrating biomass-derived carbon into the chemical industry: the left pathway illustrates decentralized biomethane production via anaerobic digestion (1G) and/or pyro-hydrothermal gasification (2G), with subsequent injection into the gas grid and transport of biogenic CO_2_ via a future CO_2_ grid to chemical plants The right pathway shows centralized biomass-to-chemicals conversion, where biomass is transported to chemical plants for on-site processing.

In addition to its chemical versatility, biomethane offers a unique infrastructural advantage: it can be seamlessly integrated into existing gas networks and utilized in conventional fossil methane-based chemical plants. In Europe, for instance, approximately 17% of biomethane production sites are connected to the high-pressure transport grid and 58% to the distribution grid, enabling a “plug-and-play” approach to defossilization. This allows biomethane to be directly substituted for fossil methane in methanol and ammonia production without requiring major capital investments. Such integration enhances supply security and reduces logistical risks associated with raw material transport. Figure 3 compares two strategic scenarios: (1) centralized biomethane production with injection (of biomethane and in the future also CO_2_) into the gas grid and (2) decentralized biomass-to-chemicals conversion at the production site. The first scenario leverages existing infrastructure and is more compatible with current industrial setups, but it may be constrained by the availability and cost of biomethane and the need for CO_2_ capture and transport infrastructure to valorize the biogenic CO_2_. The second scenario requires significant investment in on-site conversion technologies and logistics for biomass handling and storage. Moreover, many of the biomass-to-chemical routes are still at low technology readiness levels. The choice between these scenarios will depend on the economics of regional factors such as feedstock availability, infrastructure maturity, and policy incentives. For energy providers, both pathways offer strategic entry points into the sustainable carbon value chain, either by supplying biomethane and CO_2_ via existing or new networks or through the supply of low carbon hydrogen, which increases the carbon efficiency of many biomass-to-chemicals pathways, as described earlier.

Many sustainable chemical processes require large volumes of renewable electricity, either for direct electrification or for producing green hydrogen as a feedstock, raising the question of how to structure these supply chains at a global level. Verpoort et al.^46^ present a techno-economic comparison of scenarios ranging from full domestic production to the import of semi-finished products. Their analysis shows that relocating production to regions with abundant, low-cost renewable electricity can yield significant cost savings, with the greatest advantage seen when importing intermediates, e.g., urea as precursor for ammonia and methanol as precursor for ethylene and plastics. This allows regions scarce in renewable energy to retain higher-value downstream processing and keep focal jobs in the chemical industry. However, such strategies involve trade-offs, including increased reliance on international supply chains, potential carbon leakage, and the need for robust certification systems to ensure environmental integrity. For energy and chemical companies, this evolving landscape underscores the importance of aligning industrial footprints with Renewable Energy (RE) availability and developing flexible supply chain models that balance cost, resilience, and sustainability.

## Conclusions

**Figure 4:**
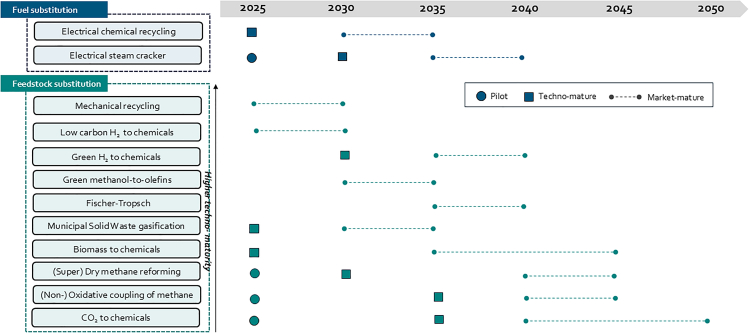
Projected development timeline and maturity levels of key technologies for fuel and feedstock substitution in the chemical industry, based on author’s synthesis Each technology is mapped across a timeline from 2025 to 2050, indicating stages of development from pilot (●), to technology mature (▪), to market mature (dotted line).

The defossilization of the chemical industry presents a unique opportunity to reconfigure the relationship between the energy and chemical sectors. While electrification of high-temperature processes has kick-started, the transition to the three sustainable carbon feedstocks (biomass, recycled plastics, and CO_2_) remains at an early stage of implementation. Given the limited availability of sustainable biomass and biogenic CO_2_, DAC is expected to become a strategic enabler of circular carbon flows, particularly in high-value applications and regions with constrained biogenic resources, even considering existing development challenges. DAC will have to overcome factors such as land availability and social acceptance alongside scaling and economical ones. However, transportation of biomass to regions with little biogenic resources also faces both technological and economic challenges. Based on the above analysis and expert iterative, Delphi-based judgment, an expected timeline from technology to market maturity is provided (Figure 4). Figure 4 is not exhaustive with respect to all technologies that exist for the production of green chemicals but focuses on those technologies that work at the interface between the energy and chemical industry. The visualization underscores the need for coordinated investment and policy support to accelerate their deployment.

Those evolutions will require a coordinated build-out of infrastructure for CO_2_ capture, purification, and distribution, as well as access to low-carbon hydrogen. The energy sector must, therefore, prepare to support not only the electrification of heat but also the development of a CO_2_-based carbon economy. For the chemical sector, this implies a dual transformation: decoupling from fossil carbon and aligning production with the geography of renewable energy and sustainable carbon availability. In this context, biomethane and biogas are likely to be valuable as feedstocks and not only as fuels, especially when leveraged through existing gas infrastructures. Hydrogen (low carbon and going forward also green) will also play a central role, not only as a clean energy carrier but also as a feedstock for carbon-rich molecules. In the near term, low-carbon hydrogen may be sufficient to initiate this transition, but long-term competitiveness will depend on the convergence of renewable energy, carbon logistics, and chemical production footprints.

Ultimately, the successful defossilization of the chemical industry will hinge on cross-sectoral collaboration, long-term policy frameworks, and strategic investment in infrastructure that links energy and chemistry. This transformation requires the combination of both technological feasibility and economic and environmental value. Driving such transformational change calls for a combination of policy interventions targeting the supply of, demand for, and innovation in these products and processes, including support for R&D and commercialization of new technologies, removal of fossil fuel subsidies that impede defossilization, and expanded GreenHouse Gas (GHG) pricing to redirect financial incentives toward low-carbon alternatives.^47^^,^^48^

While the technological pathways for a fossil-free chemicals sector are increasingly clear, their realization is constrained by political and economic realities. As Tilsted and Newell^49^ point out, petrochemicals are often ignored in global energy debates, even though they will strongly shape future energy use. Mah^50^ adds that the so-called energy revolution is complicated, because fossil fuels are not only used for energy but also as raw materials for plastics and chemicals. Shifting away from fossil-based growth will need the support of the petrochemical industry, given the political and economic influence it wields.^51^ Research on socio-technical transitions^52^ also shows that current systems are very hard to change. They are locked in by existing factories and infrastructure, government subsidies and rules, powerful industry groups, and everyday habits that people take for granted. Overcoming these barriers will, therefore, require more than just new technology. It will also need strong political and policy action, pressure from civil society to challenge entrenched interests and create space for alternatives, and institutions to support a just transition.

Linking the research questions stated in the introduction of this perspective, we could summarize our findings into the following key recommendations for policymakers and industry

(1) To what extent can electrification be a key lever to defossilize the chemical sector?
  • Recommendations: Electrify high-temperature processes for major chemicals (methanol, ammonia, and ethylene) where renewable electricity is abundantly available and grid capacity allows. Invest in grid upgrades and pilot projects to accelerate deployment. Where renewables are limited, look into the import of intermediates from energy-rich regions.
(2) What could be the role of hydrogen in the defossilization of the chemical industry?
  • Recommendations: Deploy low-carbon hydrogen, prioritizing ammonia and methanol synthesis, and integrate with CO_2_ utilization for platform chemicals. Build hydrogen infrastructure and foster cross-sector partnerships, starting with low-carbon hydrogen and transitioning to green hydrogen as renewables expand.
(3) What are the impacts of competition and opportunities for collaboration on sustainable carbon?
  • Recommendations: Invest in DAC for long-term carbon supply and integrate with renewable energy and hydrogen. Leverage existing infrastructure for biomethane and CO_2_ transport and adapt supply chains to regional realities. Align energy and chemical sector policies and support commercialization of new technologies. Encourage adaptive, regionally tailored strategies to manage uncertainty and foster collaboration.

## Acknowledgments

We are grateful to the following people for the input they gave to this study either in the form of interviews or in the form of presentations: Julia Attwood, BloombergNEF; Florie Gonsolin, Cefic; Gernot Jaeger, Covestro; Peter Roose, Eastman; Kevin Van Geem, Ghent University; Dirk Dompas, INEOS; Bert Sels, KU Leuven; Yuan-Sheng Yu, Lux Research; Johnson Magnus, Perstorp; Christopher vom Berg, Renewable Carbon Initiative; Sergio Mastroianni and Philippe Marion, Syensqo; and Bert Weckhuysen, Utrecht University.

## Declaration of interests

The authors declare no competing interests.
